# Effect of Lavender* (Lavandula angustifolia)* Essential Oil on Acute Inflammatory Response

**DOI:** 10.1155/2018/1413940

**Published:** 2018-03-18

**Authors:** Gabriel Fernando Esteves Cardia, Saulo Euclides Silva-Filho, Expedito Leite Silva, Nancy Sayuri Uchida, Heitor Augusto Otaviano Cavalcante, Larissa Laila Cassarotti, Valter Eduardo Cocco Salvadego, Ricardo Alexandre Spironello, Ciomar Aparecida Bersani-Amado, Roberto Kenji Nakamura Cuman

**Affiliations:** ^1^Department of Pharmacology and Therapeutics, State University of Maringá, Maringá, PR, Brazil; ^2^College of Health Sciences, Federal University of Grande Dourados, Dourados, MS, Brazil; ^3^Department of Chemistry, State University of Maringá, Maringá, PR, Brazil

## Abstract

*Lavandula angustifolia* is a plant of Lamiaceae family, with many therapeutic properties and biological activities, such as anticonvulsant, anxiolytic, antioxidant, anti-inflammatory, and antimicrobial activities. The aim of this study was to evaluate the effect of* Lavandula angustifolia* Mill. essential oil (LEO) on acute inflammatory response. LEO was analyzed using gas chromatography-mass spectrometry (GC-MS) and nuclear magnetic resonance spectroscopy (NMR) methods and showed predominance of 1,8-cineole (39.83%), borneol (22.63%), and camphor (22.12%). LEO at concentrations of 0.5, 1, 3, and 10 *μ*g/ml did not present* in vitro* cytotoxicity. Additionally, LEO did not stimulate the leukocyte chemotaxis* in vitro*. The LEO topical application at concentrations of 0.25, 0.5, and 1 mg/ear reduced edema formation, myeloperoxidase (MPO) activity, and nitric oxide (NO) production in croton oil-induced ear edema model. In carrageenan-induced paw edema model, LEO treatment at doses of 75, 100, and 250 mg/kg reduced edema formation, MPO activity, and NO production. In dextran-induced paw edema model, LEO at doses of 75 and 100 mg/kg reduced paw edema and MPO activity. In conclusion, LEO presented anti-inflammatory activity, and the mechanism proposed of LEO seems to be, at least in part, involving the participation of prostanoids, NO, proinflammatory cytokines, and histamine.

## 1. Introduction

The Lamiaceae family of plants is a major source of polyphenols and pharmacological properties described in the literature. Belonging to the Lamiaceae family,* Lavandula angustifolia* is indigenous to the mountainous regions of the Mediterranean, with many therapeutic properties and biological activities [1].

Phytochemical studies revealed that the major constituents of* Lavandula angustifolia* essential oil (LEO) are 1,8-cineole, camphor, and endo-borneol. Other components can also be found in minor quantities, such as *α*-pinene, camphene, *α*-pinene, *β*-pinene, p-cymene, limonene, terpinen-4-ol, and cryptone [2, 3]. However, the LEO composition may vary depending on the geographical origin of the plant material and environmental factors, such as geographical conditions, climate and seasonal variations, and the stage of the plant growth, and the extraction and detection methods also influence the LEO composition [4].

The extracts and* Lavandula angustifolia* essential oil have various pharmacological effects described in the literature, such as anticonvulsant [5], anxiolytic [6], antioxidant, anticholinesterase [7, 8], antimicrobial [9], and antifungal activities [10]. Additionally, various constituents in the oil also have valuable pharmacological properties, such as anti-inflammatory, antioxidant, and antimicrobial [11–14].

Inflammation is a complex biological process involving vascular, cellular components and a variety of soluble substances, presenting as characteristic clinical signs: redness, heat, swelling, pain, and function loss [15]. The purpose of the inflammatory process is the elimination of the aggressive agent and consequences of tissue injury [16]. The leukocytes recruitment is essential in the acute inflammatory response, where cells act as the first line of defense in the initiation of the inflammatory process, and involves the participation of several inflammatory mediators [17], produced by inflammatory cells that play an important role in maintaining the inflammatory response [18].

Natural products and their essential oils have been popularly used for the treatment of various inflammatory diseases and the development of new therapeutic strategies. Studies suggest that the use of natural products may be safer and more effective since they have low toxicity and few side effects [19]. Thus, the objective of this research was to investigate the LEO activity in acute inflammation, using different experimental models.

## 2. Materials and Methods

### 2.1. Chemicals

Zymosan, MTT [3-(4,5-dimethylthiazol-2-yl)-2,5-diphenyl-2H-tetrazolium bromide], croton oil, dextran, celecoxib, promethazine, indomethacin, and *λ*-carrageenan were purchased from Sigma-Aldrich (St. Louis, MO, USA).

### 2.2. Plant Material and Extraction of Essential Oil

The leaves and stem of the* Lavandula angustifolia* were commercially purchased from Cercopa Guarapuava, PR, Brazil. The essential oil was extracted by conventional steam distillation using a Clevenger-type apparatus for 3 h.

### 2.3. Analysis of the Essential Oil and Compound Identification

The LEO was analyzed using gas chromatography- (GC-) mass spectrometry (MS). GC was performed with a Thermo Electron Corporation Focus GC model under the following conditions: DB-5 capillary column (30 m × 0.32 mm, 0.25 mm); column temperature, 60°C (1 min) to 180°C at 3°C/min; injector temperature, 220°C; detector temperature, 220°C; split ratio, 1 : 10; carrier gas, He; flow rate, 1.0 mL/min. An injection volume of 1 *μ*L was diluted in acetone (1 : 10). The retention index (RI) was calculated relative to a series of the* n*-alkanes (C_8_–C_40_, Sigma-Aldrich, St. Louis, MO, USA) on DB-5 column, using the Van den Dool and Kratz equation [20, 21].

### 2.4. Animals

Male Swiss mice (weighing 20–30 g) were provided by the Central Animal House of the State University of Maringá, Paraná, Brazil. The animals were housed at 22 ± 2°C under a 12/12 h light/dark cycle with free access to food and water. All of the protocols were approved by the Ethical Committee in Animal Experimentation of the State University of Maringá (CEEA/UEM number 3024210315).

### 2.5. Cell Viability Analysis (MTT Assay)

Leukocytes were obtained from the peritoneal cavity of mice 4 hours after injection of zymosan solutions (1 mg/cavity, i.p.). Briefly, the cells (5 × 10^5^ cells/well) were exposed to LEO at concentrations of 0.5, 1, 3, 10, 30, or 90 *μ*g/mL for 90 min (37°C/CO_2_ 5%). A volume of 10 *μ*L of MTT (5 mg/mL, Sigma) was added to each well. After 2 h, 150 *μ*L of supernatant was removed, and 100 *μ*l of dimethyl sulfoxide was added to each well, and absorbance was measured using a Biochrom Asys Expert plus microplate reader (Asys®) at a wavelength of 540 nm, as previously described [22]. The percentage of viability was determined by the following formula:

(1) %  viable  cells=absorbance  of  the  treated  cells−absorbance  of  the  blankabsorbance  of  the  control−absorbance  of  the  control×100.

### 2.6. *In Vitro* Leukocytes Chemotaxis

Leukocytes were obtained by the method described above. The chemotaxis assay was performed using a 48-well microchemotaxis plate (Neuro Probe), in which the chambers were separated by a polyvinylpyrrolidone-free polycarbonate membrane (5 *μ*m pore size). LEO (used as chemoattractant) at concentrations of 2, 15, or 150 *μ*g/mL or RPMI 1640 medium (control) was placed in the lower chamber. A leukocyte suspension (1 × 10^6^ cells/mL, in RPMI 1640 medium) was placed in the upper chamber. The chambers were incubated (37°C/CO_2_ 5%) for 1 h and the membrane was stained with Instant Prov. Cells present in the membrane were counted in five random fields from each well, using light microscopy, as previously described [22]. The results are expressed as the mean number of leukocytes per field.

### 2.7. Evaluation of Topical Anti-Inflammatory Effect

Ear edema was induced by topical application of croton oil (200 *μ*g 20/ear) diluted in 20 *μ*l of acetone/water solution (vehicle) in the inner surface of the mouse right ear. The left ear received an equal volume of vehicle (*n* = 5–7 animals/group). LEO (0.125, 0.25, 0.5, 1, and 2.5 mg/ear), dexamethasone (reference drug, 0.1 mg/ear), or vehicle was applied topically to the right ear 1 h before croton oil application. Six hours after application of the inflammatory stimulus, the mice were euthanized, and a 6 mm diameter plug was removed from both the treated and untreated ears. Edema was measured as the weight difference between the two plugs. The data are expressed as the mean ± SEM weight of the ears.

### 2.8. Evaluation of Systemic Anti-Inflammatory Effect

To provide additional evidence supporting the potential anti-inflammatory effects produced by LEO, we also carried out a carrageenan or dextran-induced mice paw edema in mice (*n* = 5–7 animals/group). The negative control group received only subplantar injection of sterile saline. The positive control group received subplantar injection of carrageenan or dextran (500 *μ*g/paw) and only treatment orally with LEO (50, 75, 100, and 250 mg/kg). The paw volume was measured by digital plethysmometer (Ugo Basile®, Italy) prior, 1, 2, and 4 hours after carrageenan injection, or 30, 60, 120, and 240 minutes after dextran injection. Indomethacin (5 mg/kg, p.o.) and celecoxib (10 mg/kg, p.o.) were used as the reference drug in carrageenan-induced foot paw edema, and promethazine (10 mg/kg, p.o.) was used as the reference drug in dextran-induced paw edema. The paw edema, in *μ*L, was calculated by the difference in the paw volume prior and after carrageenan or dextran injection. After the last measurement, the animals were euthanized and the inflamed hind paws tissues were collected.

### 2.9. Determination of Myeloperoxidase (MPO) Activity

The plugs obtained from the right and left ears and paw sections were used to analyze myeloperoxidase (MPO) activity. The ear and paws sections were placed in 50 mM potassium phosphate buffer (pH 6.0) that contained 0.5% hexadecyl trimethyl ammonium bromide (Sigma, St. Louis, MO, USA) in a Potter homogenizer. The homogenate was shaken and centrifuged for 5 min. A 10 *μ*L aliquot of the supernatant was added in triplicate to each well of microplate, in triplicate. The supernatant solution was then mixed with 200 *μ*L of the buffer solution that contained* O*-dianisidine dihydrochloride (16.7 mg, Sigma), double distilled water (90 mL), potassium phosphate buffer (10 mL), and 1% H_2_O_2_ (50 *μ*L). The enzyme reaction was stopped by addition of sodium acetate. MPO activity was determined by the absorbance measured at 460 nm using a microplate spectrophotometer (Spectra Max Plus).

### 2.10. Determination of Nitric Oxide (NO) Production

The NO production was determined by measuring the nitrite level by Griess reaction. Nitrite level was determined in ears and paws sections, obtained as above described. The samples were centrifuged at 1000 rpm for 10 min at 4°C. The supernatant was separated (50 *μ*L) and incubated with equal volumes of Griess reagent mixtures (1% sulfanilamide in 5% phosphoric acid and 0,1% * N*-1-naphthylethylenediamine dihydrochloride in water) at room temperature for 10 min. The absorbance was measured in a microplate reader at 550 nm. NO concentrations were calculated from a sodium nitrite standard curve. Data were presented as the *μ*M concentration of NO^2-^.

### 2.11. Statistical Analysis

Data are expressed as the mean ± SEM for each experimental group. The results were statistically analyzed by using one-way variance analysis (ANOVA) followed by Tukey's test. Differences were considered significant when* p* < 0.05.

## 3. Results

### 3.1. Analysis of LEO

**Figure 1:**
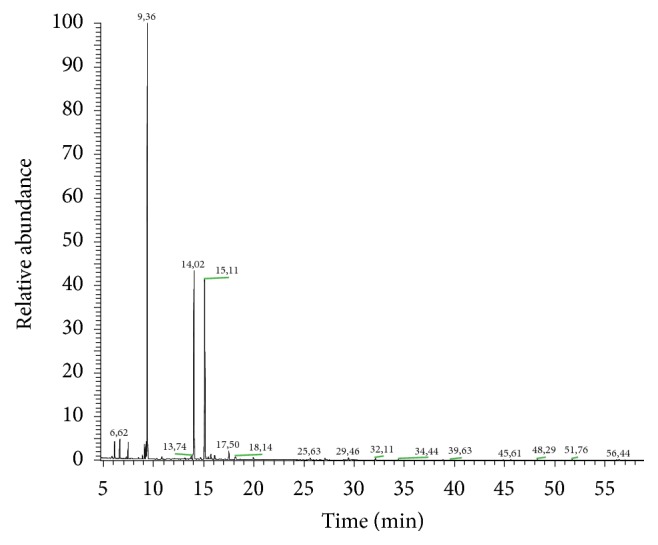
GC chromatogram of the* Lavandula angustifolia* essential oil. The identified peaks are attributed to majority compounds: 1,8-cineole (9.36); camphor (14.02); borneol (15.11).

The obtained pale yellow essential oil was dried over sodium sulfate and stored at 4°C in dark vials until tested. The yield of LEO was 0,14% v/w. The chemical composition of LEO was investigated by gas chromatography-mass spectrometry (GC-MS). The results of the GC-MS analysis (Figure 1) showed a predominance of 1,8-cineole (39,8%), endo-borneol (22,6%), and camphor (22,1%). A complete list of the components and their relative abundances are presented in Table 1.

### 3.2. Cell Viability

In the cell viability assay, LEO at concentrations of 0.5, 1, 3, 10, 30, and 90 *μ*g/ml presented cell viability of 79, 77, 76, 76, 68, and 60%, respectively. Our data indicate that LEO has low cytotoxicity* in vitro* at low concentrations, with cell viability greater than 75% up to a concentration of 10 *μ*g/mL.

### 3.3. *In Vitro* Chemotactic Effect of LEO

The present study evaluated the chemotactic effects of LEO at different concentrations (2, 15, and 150 *μ*g/ml) on leukocyte chemotaxis* in vitro*. However, LEO did not increase leukocytes chemotaxis in any concentrations when compared to the vehicle (RPMI-1640). The fMLP (10^-6 ^M) (positive control) induced a significant leukocyte migration (data not shown).

### 3.4. Effects of LEO on the Topical Inflammation Induced by Croton Oil

**Figure 2:**
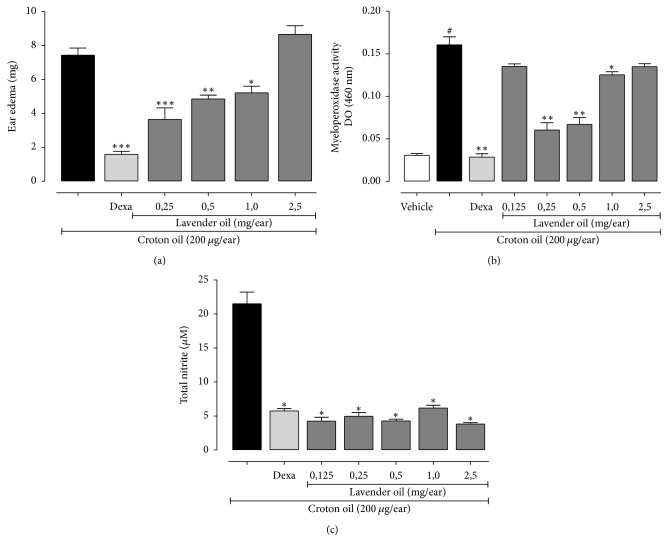
The animals were treated topically with LEO (0.125, 0.25, 0.5, 1.0, and 2.5 mg/ear) or Dexa (0.1 mg/ear) 1 h before the croton oil-induced ear edema, ^*∗*^*p* < 0.05, ^*∗∗*^*p* < 0.001, and ^*∗∗∗*^*p* < 0.0001, compared with croton oil (a). Myeloperoxidase enzyme activity, ^*∗*^*p* < 0.001, ^*∗∗*^*p* < 0.0001 compared with croton oil and ^#^*p* < 0.0001 compared to vehicle (Vh) (b). Nitric oxide levels, ^*∗*^*p* < 0.0001 compared with croton oil (c). The ear edema, the activity of myeloperoxidase, and the nitric oxide concentration were determined 6 hours after application of croton oil. Data are expressed as mean ± SEM (one-way ANOVA, Tukey test).

The topical effect of LEO on ear edema induced by croton oil was demonstrated. Topical pretreatment with LEO (0.25, 0.5, and 1,0 mg/ear) reduced ear edema induced by croton oil by 59.6, 36.3, and 30.6%, respectively. Topical pretreatment with dexamethasone (0.1 mg/ear) (reference drug) reduced ear edema by 78.7% (Figure 2(a)).

The effects of LEO on MPO activity in ears tissues were also demonstrated. The activity of MPO was decreased in the group treated topically with LEO at concentrations of 0.25, 0.5, and 1.0 mg by 62.5%, 58.3, and 21.8%, respectively, compared with the control group. Dexamethasone reduced MPO activity by 82.5% (Figure 2(b)).

The nitrite levels were used as parameters to evaluate the NO production in ears sections after croton oil-induced edema. The nitrite level increased significantly in the edema ear 6 h after croton oil topical application. Pretreatment with LEO (0.125, 0.25, 0.5, 1.0, 2.5, and 5.0 mg/ear) promoted a decrease in the nitrite levels by 80, 76, 80.2, 71.3, 82.3, and 81.3%, respectively. Dexamethasone also reduced nitrite level by 73.2% (Figure 2(c)).

### 3.5. Carrageenan-Induced Paw Edema in Mice

**Figure 3:**
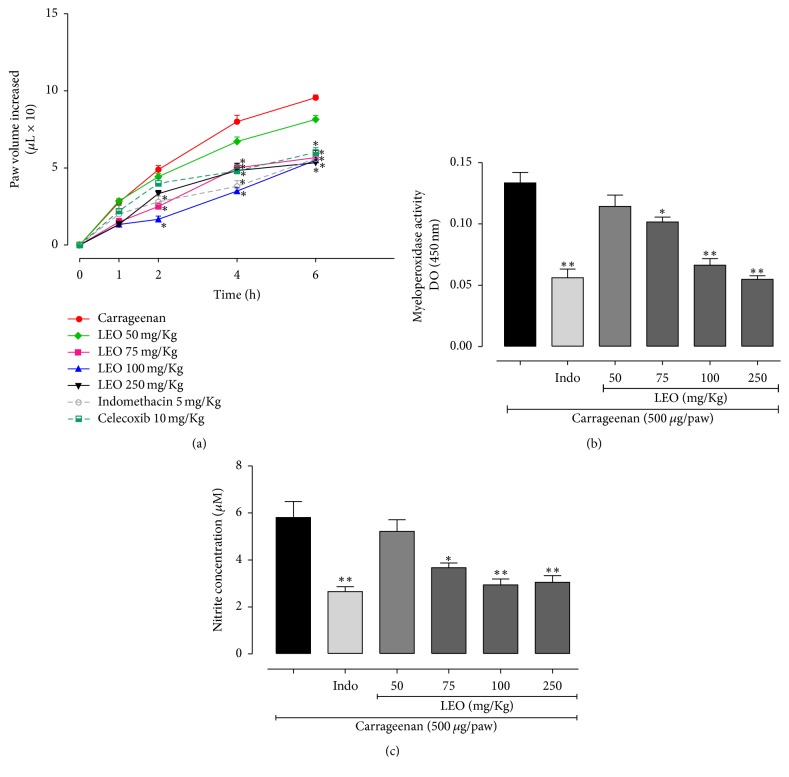
Effect of the treatment with LEO on carrageenan-induced paw edema in mice (a). Myeloperoxidase activity (b). Nitric oxide concentration (c). Values represent mean values ± standard error of the mean for each group. A significant difference at ^*∗*^*p* < 0.001 and ^*∗∗*^*p* < 0.0001 compared with the carrageenan (Cg) group (one-way ANOVA, Tukey test).

**Figure 4:**
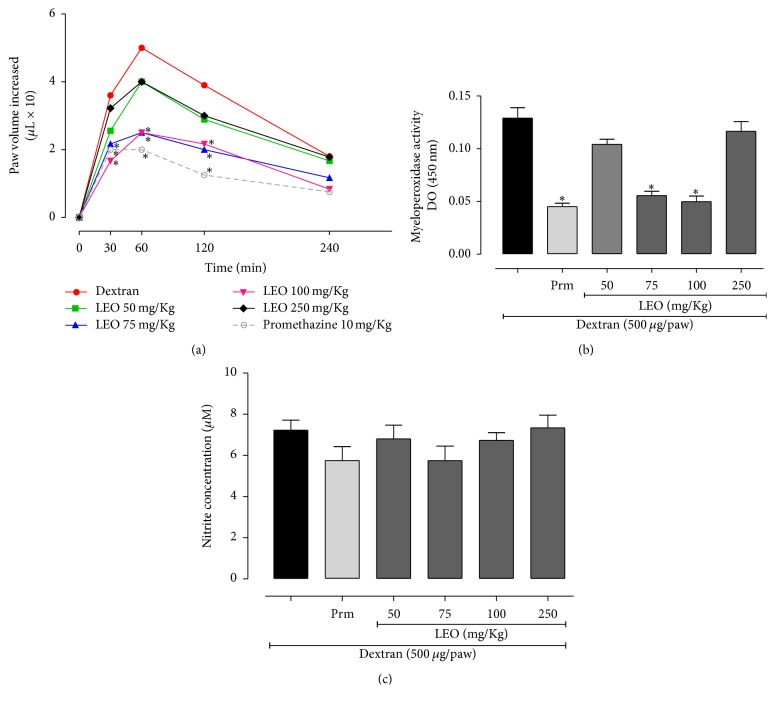
Effect of the treatment with LEO on dextran-induced paw edema in mice (a). Myeloperoxidase activity (b). Nitric oxide concentration (c). Values represent mean values ± standard error of the mean for each group. A significant difference at ^*∗*^*p* < 0.001, compared with the carrageenan (Cg) group (one-way ANOVA, Tukey test).

The subcutaneous injection of carrageenan in the mouse paw promoted a local inflammatory response with edema peak in 6 h after carrageenan injection. As shown in Figure 4(a), when compared with positive control group, the treatment with LEO at 75 and 100 mg/kg significantly reduced the development of edema 2, 4, and 6 h after carrageenan injection, and LEO at dose of 75 mg/kg reduced paw edema by 48.7, 37.5, and 40.7%, respectively. At dose of 100 mg/kg, LEO treatment reduced paw edema by 65.7, 56.2, and 42.4%, respectively. The treatment with LEO at dose of 250 mg/kg significantly reduced the development of edema in 4 and 6 h after carrageenan injection by 39.6% and 44.1%, respectively. The treatment with indomethacin (reference drug) reduced the development of edema 2, 4, and 6 h after carrageenan injection by 42.3, 52.5, and 41.3%, respectively, and celecoxib (reference drug) reduced the development of edema 4 and 6 h after carrageenan injection by 40% and 37.18 (Figure 3(a)).

The MPO enzyme activity was evaluated 6 h after edema induction. The treatment with LEO at doses of 75, 100, and 250 mg/kg significantly reduced the activity of MPO in 25%, 50.3%, and 59.4%, respectively, similar to indomethacin (57.1%) (Figure 3(b)).

Additionally, the concentration of nitrite levels was evaluated. The treatment with LEO at doses of 75, 100, and 200 mg/kg reduced significantly the nitrite levels by 36.7, 49.4, and 47.6%, respectively, and a similar effect was observed with indomethacin (reference drug) treatment, with 54.3% of reduction (Figure 3(c)).

### 3.6. Dextran-Induced Paw Edema in Mice

The subcutaneous injection of dextran in the mouse paw caused a local inflammatory response with edema peak in the 1 h after injection. As shown in Figure 4(a), the treatment with LEO at 75 and 100 mg/kg significantly reduced the edema development in 30, 60, and 120 min after dextran injection, compared with control group. LEO treatment at dose of 75 mg/kg reduced edema by 40, 50, and 48.7%, at times of 30, 60, and 120 min, respectively, and at dose of 100 mg/kg, LEO treatment reduced paw edema by 53.8, 56.2, and 44.6%, respectively. Promethazine (reference drug) reduced the edema development at 30, 60, and 120 min, by 45.4, 60, and 67.9%, respectively (Figure 4(a)).

The LEO treatment at doses of 75 and 100 mg/kg reduced significantly MPO activity by 57.4% and 62%, respectively, similar to effect observed with promethazine (65.1% of reduction). The LEO treatment at 50 and 250 mg/kg did not reduce significantly the MPO activity (Figure 4(b)). LEO treatment did not significantly reduce the nitrite levels in paw edema induced by dextran (Figure 4(c)).

## 4. Discussion

As with the use of any essential oil, there is care about possible allergic reactions or irritation of the skin with the use of lavender. The topical use of lavender is very common in perfumes, cosmetics, and cleaning products [23]. It is well reported that lavender has been frequently responsible for dermatitis [24, 25] and reactions to the sensitivity of other members of the Lamiaceae family [26]. It can be evidenced by the topical application of LEO in the highest concentrations, where an absence of anti-inflammatory effect was observed.

*Lavandula angustifolia* Mill. is characteristic of strong fragrance, indicating that there are rich aromatic compounds. The essential oil obtained from the leaves and stem is rich in monoterpene and phenols, and the GC-MS analysis showed that a predominance was due to 1,8-cineole, borneol, and camphor. The total amount of each of them was high, which suggests that the three chemical constituents in* Lavandula angustifolia* Mill. leaves may play major roles in the biological activities and pharmacological properties. Another phytochemical study with this plant revealed the presence of the same major constituents of LEO, however, in different concentrations [2]. The chemical composition of essential oils may show variations due to geographic conditions, climate, seasonality, and extraction methods [4, 27].

In the literature, there is a great variety of studies demonstrating the activity of essential oils and compounds containing monoterpenes and sesquiterpenes, on the acute inflammatory response and immunomodulatory activity, for example, camphor, estragole, anethole,* Citrus latifolia* essential oil, and* Pogostemon cablin* essential oil, in models involving leukocyte chemotaxis, edema formation, cellular recruitment, and phagocytosis [12, 22, 28–30].

In the cell viability assay, it was found that LEO in high concentrations (30 and 90 ug/ml) affected cell viability. However, at lower concentrations LEO was not proved to be cytotoxic. Confirming our results, a study reported that the essential oil of lavender has the cytotoxicity dose-dependent manner and can vary with its constituents [3]. In a study performed by Alnamer et al. [31] it was observed that the oral administration of extract of* Lavandula officinalis* is not toxic and does not cause significant changes in the body weight. This result indicates that the use in low concentrations can be considered safe.

The present study also evaluated a possible irritative effect of LEO at different concentrations on leukocyte chemotaxis* in vitro*. The results showed that LEO did not stimulate the leukocyte chemotaxis, indicating the absence of a leukocyte migration stimulating effect of LEO.

In ear edema induced by croton oil, LEO reduced edema formation, MPO activity, and nitrite levels, but in high concentrations (2.5 mg/ear) this effect is not observed, and LEO did not reduce edema formation but rather presented an irritative response, increasing edema by 16.5% compared to croton oil. Several studies demonstrated that the lavender has an irritating effect after their exposure to the skin [32, 33]. The constituents present in the essential oil of lavender oxidize when exposed to air and can cause skin irritation [34]. Under such conditions, the topical use of lavender in perfumes, cosmetics, and cleaning products should be done with caution.

The croton oil is a phlogistic agent that induces an inflammatory response by activating phospholipase A_2_ and initiating arachidonic acid metabolites involved in edema and leukocyte migration is associated with alterations in cytokine production and increased production of prostaglandins and leukotrienes [35]. Studies demonstrated anti-inflammatory activity of 1,8-cineole (as major constituent of LEO) by inhibition of cytokine production (such as tumor necrosis factor (TNF) and interleukin- (IL-) 1*β*) and arachidonic acid metabolism [36] and decrease of TNF and IL-1*β* production, nuclear factor kappa B (NF-*κ*B), and toll-like receptor 4 (TLR4) expression, and MPO activity in lung tissue in LPS-induced acute pulmonary inflammation in mice [37]. It has also been demonstrated that systemic administration of camphor (one of the major constituents of LEO) reduced the edema formation and MPO activity in croton oil-induced ear edema model [12]. Thus, the anti-inflammatory effect of LEO may be attributable to a single or synergistic effect of its main components.

MPO is an indirect marker of neutrophil infiltration into tissue. Decreases in MPO activity suggest less neutrophil infiltration [38]. Studies with essential oils, monoterpenes, and sesquiterpenes have demonstrated reduction of MPO activity [12, 39, 40]. In our study, the topical application of LEO reduced ear edema induced by croton oil, MPO activity, and NO levels, and a similar effect was observed with dexamethasone (reference drug). Based on our results, we suggest that topical treatment with LEO reduced leukocytes infiltration (observed in MPO activity) and it could be involved in the decrease of the NO production, as observed, and decrease of arachidonic acid metabolites.

Nitric oxide (NO) is a signaling molecule that plays a key role in the pathogenesis of inflammation; NO may exhibit an anti-inflammatory effect under normal physiological conditions; however, it may play a proinflammatory role because of its excessive production in abnormal situations. NO has an important role in infection control, leukocyte migration, and cytokine production [41, 42]. In our study, topical treatment with LEO reduced NO production. It has been shown that treatment of PC_12_ cells with LEO reduced the NO, TNF, IL-1*β*, and IL-6 production in *β* amyloid peptide (A *β*) induced inflammation [43].

The development of the inflammatory response induced by carrageenan is characterized by two different phases: an initial stage (1-2 h) which is dependent on the release of histamine, serotonin, and bradykinin, followed by a later stage (3-4 h) which is primarily maintained by the release of prostanoids and NO in tissue [44, 45]. It was also observed that, in addition to these mediators, there is an increased production of proinflammatory cytokines, such as TNF, IL-1*β*, and IL-6 [46].

A study demonstrated that 1,8-cineole (a major constituent of LEO) reduced edema formation in carrageenan-induced paw edema model in rats and mice, and the proposed mechanism was the reduction of prostaglandins and proinflammatory cytokines [47]. In another study, it was demonstrated that the* Artemisia argyi* essential oil (containing camphor and borneol among the main constituents) inhibited edema formation in carrageenan-induced paw edema in rats [48]. Thus, we suggest that anti-inflammatory activity of LEO could be related to 1,8-cineole, borneol, and camphor, found as major constituents of this oil, and also to the other constituents.

In our work, LEO at doses of 75 and 100 mg/kg inhibited edema formation already in first phase of carrageenan effect showing similar anti-inflammatory effects to COX antagonist (indomethacin, nonsteroidal anti-inflammatory, used as reference drug) and only dose of 250 mg/kg LEO treatment inhibited edema formation in the second phase of carrageenan induction with a similar effect to COX-2 selective antagonist (celecoxib, nonsteroidal anti-inflammatory, used as reference drug). COX-2, an inducible enzyme found in activated inflammatory cells, plays a crucial role in cytokine production and prostanoid mediator release. The inhibition of COX-2 protein expression has been used to evaluate the anti-inflammatory effects of compounds* in vivo* and* in vitro* [49, 50]. These data show that possibly the mechanism of action of LEO is involved with inactivation of COX in carrageenan-induced paw edema.

Additionally, LEO treatment decreased neutrophil infiltration (MPO activity) in a dose-dependent manner and NO production. We suggest that the anti-inflammatory activity of LEO could be, in part, involved with reduced prostanoids, proinflammatory cytokines, and NO production. In addition, the essential oil may be interfering with the production or release of vasoactive amines, such as serotonin and histamine.

We also evaluated LEO on dextran-induced paw edema. Dextran is a high molecular weight polysaccharide, which, different to carrageenan, induces anaphylactic reactions characterized by extravasation and formation of edema which can be detected within the first 30 min after induction, due to mastocyte degranulation with release of histamine, serotonin, and other mediators [51]. In our work, LEO treatment at doses of 75 and 100 mg/kg reduced paw edema induced by dextran and MPO activity, suggesting decrease of neutrophil infiltration, and a similar effect was observed with promethazine (antihistaminic used as reference drug). This response could be attributable to the release of different autacoids, including histamine. Essential oils can promote the release of histamine and other mediators, acting as irritative agents [52].

NO is released under basal conditions and its production can be markedly stimulated by bradykinin, acetylcholine, and particularly histamine [53]. The receptor involved in nitric oxide release evoked by histamine must be of the H1, histaminergic subtype [54]. In this model LEO treatment did not show effect on NO production, indicating the LEO mechanism is probably not involved with H1 receptor. It has been demonstrated that* Artemisia iwayomogi* extract, containing camphor and borneol (among the main constituents of LEO), inhibited compound 48/80–induced systemic reactions in mice and attenuated histamine release from rat peritoneal mast cells activated by compound 48/80 or IgE [55]. Furthermore, Kim and Cho [56] demonstrated that the essential oil of lavender, applied topically, inhibits histamine release from peritoneal mast cells induced by compound 48-80. Based on our results, we suggest that LEO may inhibit the release of histamine by mast cells.

## 5. Conclusion

Our results suggest that LEO has antiedematogenic activity and possesses an anti-inflammatory activity, both in the topical treatment and orally. The mechanism proposed of LEO seems to be, at least in part, involving the participation of prostanoids, proinflammatory cytokines, NO, and histamine. LEO affects inflammatory response and exerts anti-inflammatory effects at low doses but has an irritant effect at higher doses. Further studies are needed to elucidate the mechanism of the action of LEO.

## Figures and Tables

**Table 1:** Chemical composition of essential oil of LEO.

RI^a^	Compounds	% RA^b^	Identification methods^c^
922	*α*-Thujene	0.15	(MS, RI)^*∗*^
932	*α*-Pinene	1.26	MS, RI
948	Camphene	1.50	MS, RI
971	Sabinense	0.23	MS, RI
977	*β*-Pinene	1.44	MS, RI
988	*α*-Phellandrene	0.17	MS, RI
1008	*δ*-3-Carene	0.17	(MS, RI)^*∗*^
1019	*p*-Cymene	0.37	MS, RI
1024	*o*-Cymene	1.45	(MS, RI)^*∗*^
1028	Limonene	1.62	MS, RI
1031	1,8-Cineole	39.83	MS, RI
1069	*cis*-Sabinene hydrate	0.31	(MS, RI)^*∗*^
1126	*α*-Camphonelal	0.15	(MS, RI)^*∗*^
1140	*trans*-Pinocarveol	0.22	(MS, RI)^*∗*^
1147	Camphor	22.12	MS, RI
1163	Pinocarvone	0.42	(MS, RI)^*∗*^
1172	Borneol	22.63	MS, RI
1180	Terpinen-4-ol	0.31	MS, RI
1186	Cryptone	0.72	(MS, RI)^*∗*^
1195	Dihydrocarveol	0.56	(MS, RI)^*∗*^
1227	*cis*-Sabinene hydrate acetate	1.12	(MS, RI)^*∗*^
1242	Cuminaldeyde	0.60	MS, RI
1283	Bornyl acetate	0.31	(MS, RI)^*∗*^
1416	*β*-Caryophyllene	0.23	MS, RI
1452	E-*β*-Farnesene	0.21	MS, RI
1510	*γ*-Cadinene	0.27	(MS, RI)^*∗*^
1578	Caryophyllene oxide	0.17	MS, RI
—	Other minor compounds	1.06	MS, RI

^a^RI: retention index, obtained with reference to *n*-alkane series C_8_H_18_–C_20_H_42_ on DB-5 column, using the van Den Dool and Kratz equation [20].   ^b^Relative area (peak area relative to the total peak area). ^c^Identification based on retention index (RI) and mass spectra (MS) of authentic compounds. ^*∗*^Identification based on the literature.
